# Effects of GLP-1RA and SGLT2i, Alone or in Combination, on Mouse Models of Type 2 Diabetes Representing Different Disease Stages

**DOI:** 10.3390/ijms222111463

**Published:** 2021-10-25

**Authors:** Masao Koike, Hitoki Saito, Genta Kohno, Masahiro Takubo, Kentaro Watanabe, Hisamitsu Ishihara

**Affiliations:** Division of Diabetes and Metabolic Diseases, Nihon University School of Medicine, 30-1 Oyaguchi-kamicho, Itabashi-ku, Tokyo 173-8610, Japan; koike.masao@nihon-u.ac.jp (M.K.); saito.hitoki@nihon-u.ac.jp (H.S.); kohno.genta@nihon-u.ac.jp (G.K.); takubo.masahiro@nihon-u.ac.jp (M.T.); watanabe.kentaro@nihon-u.ac.jp (K.W.)

**Keywords:** type 2 diabetes mellitus, GLP-1 receptor agonists, SGLT2 inhibitors, pancreatic β-cells, fatty liver

## Abstract

Glucagon-like peptide-1 receptor agonist (GLP-1RA) and sodium-dependent glucose transporter 2 inhibitor (SGLT2i), in addition to lowering glucose, have pleiotropic effects on the heart, kidneys, and liver. These drugs have thus come into widespread use for treating type 2 diabetes (T2DM). However, mechanistic comparisons and effects of combining these drugs have not been adequately studied. Employing diet-induced obese (DIO) mice and db/db mice as models of the early and advanced stages of T2DM, we evaluated effects of single or combined use of liraglutide (a GLP-1RA) and ipragliflozin (a SGLT2i). Treatments with liraglutide and/or ipragliflozin for 28 days improved glycemic control and reduced hepatic lipid accumulation similarly in DIO mice. In contrast, in db/db mice, despite similar favorable effects on fatty liver, liraglutide exerted no beneficial effects on glycemic control. Improved glycemic control in db/db mice treated with ipragliflozin was accompanied by increased pancreatic β-cell area and insulin content, both of which tended to rise further when ipragliflozin was combined with liraglutide. Our data suggest that liraglutide is more efficient at an earlier stage and ipragliflozin can be effective in both stages. In addition, their combined use is a potential option for treating advanced stage diabetes with fatty liver disease.

## 1. Introduction

Diabetes mellitus represents a leading clinical challenge worldwide. There are eight classes of oral drugs for diabetes (biguanides, sulfonylureas, glinides, α-glucosidase inhibitors, thiazolidinediones, DPP-4 inhibitors, and sodium-dependent glucose transporter 2 inhibitors (SGLT2i)) and two varieties of injectable agents (insulins and glucagon such as peptide-1 (GLP-1) receptor agonists (GLP-1RAs)). Now, semaglutide, a GLP-1RA, can be orally administered. Among these drugs, GLP-1RAs and SGLT2is are attracting much attention, since recent clinical trials revealed that cardiovascular and renal complications can be prevented with the administration of GLP-1RAs [1] and SGLT2is [2]. Beneficial effects are also observed in patients with fatty liver disease [3]. GLP-1RAs act on pancreatic β-cells, augmenting insulin secretion. These drugs also suppress appetite and are beneficial for long term weight management [4]. SGLT2is, on the other hand, directly reduce energy expenditure by forced excretion of glucose into urine, releasing glucose toxicity on β-cells as well as peripheral tissues. Based on the results of large clinical trials, the American Diabetes Association and the European Association for the Study of Diabetes have recommended the use of GLP-1RA and SGLT2i, especially in patients with cardiovascular risks and/or complications [5]. Furthermore, effects of GLP-1RAs and SGLT2is are mutually compensatory, because GLP-1RAs exert anti-inflammatory effects while SGLT2is have beneficial effects on hemodynamics. SGLT2is often cause hyperphagia due to excessive glucose loss in the urine, which is contrary to the appetite suppressing effect of GLP-1RAs. Therefore, combined therapy with these agents might be an advanced option [6] for glycemic control, and more specifically, for treating diabetes with fatty liver disease [7,8].

In addition to choices of glucose lowering agents, the appropriate timing of drug administration is also important. Pathophysiology and thus drug effects differ among stages of type 2 diabetes mellitus (T2DM). Generally, T2DM development is proceeded by rising insulin resistance. The increased insulin resistance then leads to a mild elevation of blood glucose, which results in glucose toxicity to pancreatic β-cells, suppressing β-cell insulin secretory function and exacerbating blood glucose elevation [9]. In the advanced stage, β-cell function and β-cell survival is compromised. GLP-1 and GLP-1RAs augment endogenous insulin secretion. Thus, if the insulin production in β-cells is suppressed by glucose toxicity or β-cell numbers are reduced, GLP-1RAs cannot exert their effects. SGLT2 inhibitors ameliorate rises in blood glucose levels by accelerating urinary glucose excretion. Their effects are independent of insulin action, and blood glucose can therefore be reduced in patients with severe impairment of endogenous insulin production, even those with type 1 diabetes [10]. 

In this study, in order to gain insight into the effects of GLP-1RAs, SGLT2is, and their combined use in different stages of diabetes, we analyzed diet-induced diabetes (DIO) mice as an early stage diabetes model and leptin receptor deficient C57BL/6^+Lepr<db>/+Lepr<db>^ (db/db) mice as an advanced stage diabetes model. Both murine models were treated with these agents individually and in combination. Herein, we focused on the effects of these drugs on β-cells and the liver.

## 2. Results

### 2.1. Effects on Body Weight and Blood Glucose in DIO Mice

C57BL/6J mice were fed a 60% high fat diet (HFD) starting at 4 weeks of age to generate DIO mice, a model of T2DM. Drug treatment was started at 16 and continued until 20 weeks of age, with a similar 60% HFD. There were four groups of DIO mice: LIRA (rated with liraglutide alone), IPRA (ipragliflozin alone), Combo (liraglutide plus ipragliflozin), and Controls (vehicles).

**Figure 1:**
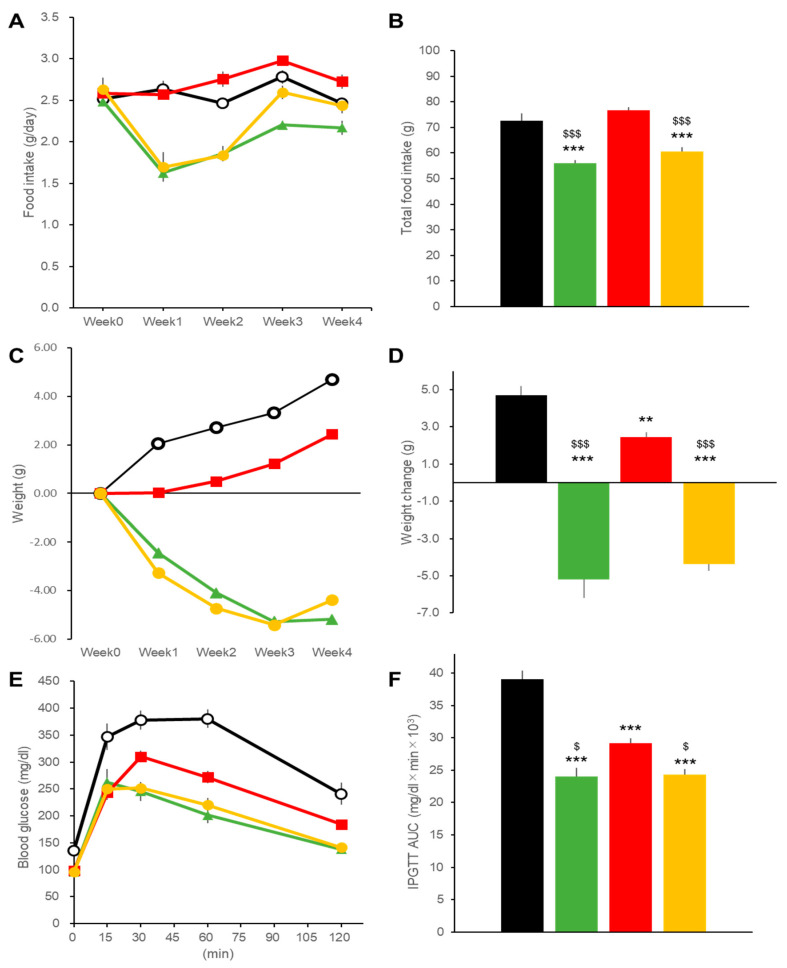
Effects of treatments with liraglutide and/or ipragliflozin on food intakes, body weight, and glycemic control in DIO mice. Time course (**A**) and total amounts (**B**) of food intake in vehicle-treated DIO mice (black line and bar) and in mice treated with liraglutide (green), ipragliflozin (red), and the combination regimen (orange). Time courses (**C**) and final changes (**D**) of body weight in mice treated with liraglutide and/or ipragliflozin. Blood glucose excursions during intraperitoneal glucose tolerance tests (**E**) and AUC (**F**) calculated from (**C**). Data represent mean ± SE, *n* = 6 DIO mice for four different treatments. ** *p* < 0.01, *** *p* < 0.001 vs. Control; $ *p* < 0.05, $$$ *p* < 0.001 vs. IPRA group. Week0 was 16 weeks of age.

Daily food intakes, around 2.5 g/day, decreased in the DIO mice in the LIRA and Combo groups with a tendency for recovery at 15 weeks in the LIRA group and complete recovery in mice receiving the combination regimen (Figure 1A,B). Body weights at the baseline, 34.4 ± 0.6 g (mean ± SEM, *n* = 24), were reduced in the LIRA and Combo groups after 4- week treatments (Figure 1C). The IPRA group showed increased body weights as compared to the baseline (Figure 1C), but the body weight increases were significantly lower than those in the Control group (Figure 1D). 

Glycemic excursions during an intraperitoneal glucose tolerance test (iPGTT) were reduced by all treatment regimens as indicated by the glucose area under the curve for 120 min (AUC120) (Figure 1E,F). Ipragliflozin treatment was less efficient at controlling glucose levels than either liraglutide alone or liraglutide plus ipragliflozin (Figure 1F).

### 2.2. Pancreatic Effects in DIO Mice

**Figure 2:**
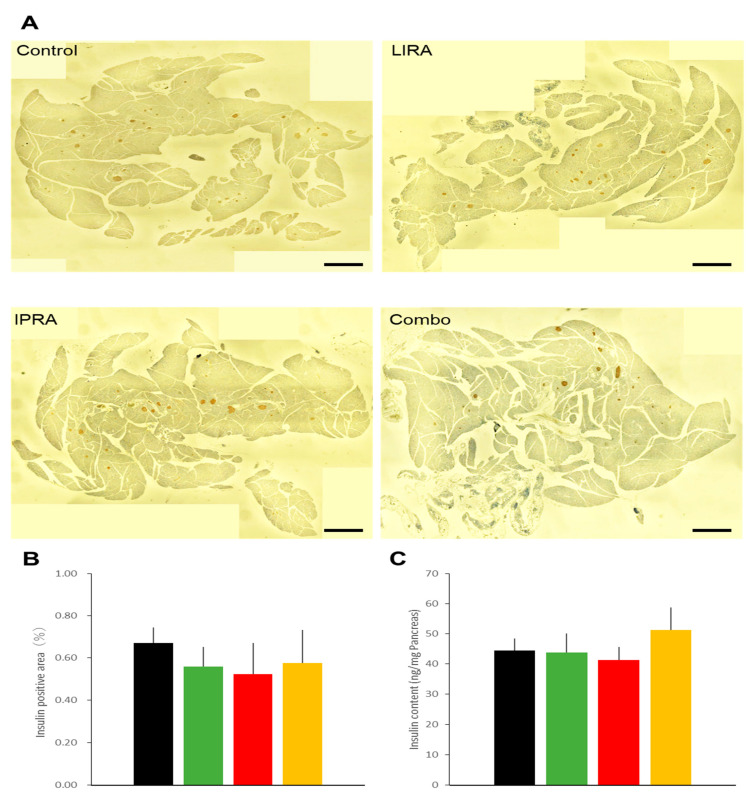
Effects of treatments with liraglutide and/or ipragliflozin on pancreatic islets in DIO mice. Representative pancreatic sections (**A**), insulin positive areas (**B**), and insulin contents (**C**) of vehicle-treated DIO mice and those treated with liraglutide and/or ipragliflozin. *n* = 4~5 DIO mice per group. Scale bars = 500 μm. In addition to data from the mice presented in other experiments, another cohort was established to examine the pancreatic effects of these interventions, since insulin positive areas and insulin content cannot be obtained simultaneously from one mouse. Effects on glycemic control in this additional cohort did not differ from other cohorts.

Fasting plasma insulin and glucagon levels were similar in the mice receiving the four different treatment regimens (Table 1). There were no differences in islet insulin positive areas (Figure 2A,B) and pancreatic insulin content among the four groups (Figure 2C). 

### 2.3. Hepatic Effects in DIO Mice

While plasma aspartate aminotransferase (AST) levels were similar, alanine aminotransferase (ALT) concentrations tended to be lower in the IPRA group and significantly lower in the LIRA and Combo groups than in the Control group (Table 1). Plasma cholesterol was slightly but significantly lower in the IPRA than in the Control group and was further reduced in the LIRA and Combo groups. Plasma triglyceride (TG) levels were slightly lower in the IPRA group and Combo groups than in the Control group, but the differences failed to reach statistical significance (Table 1).

**Figure 3:**
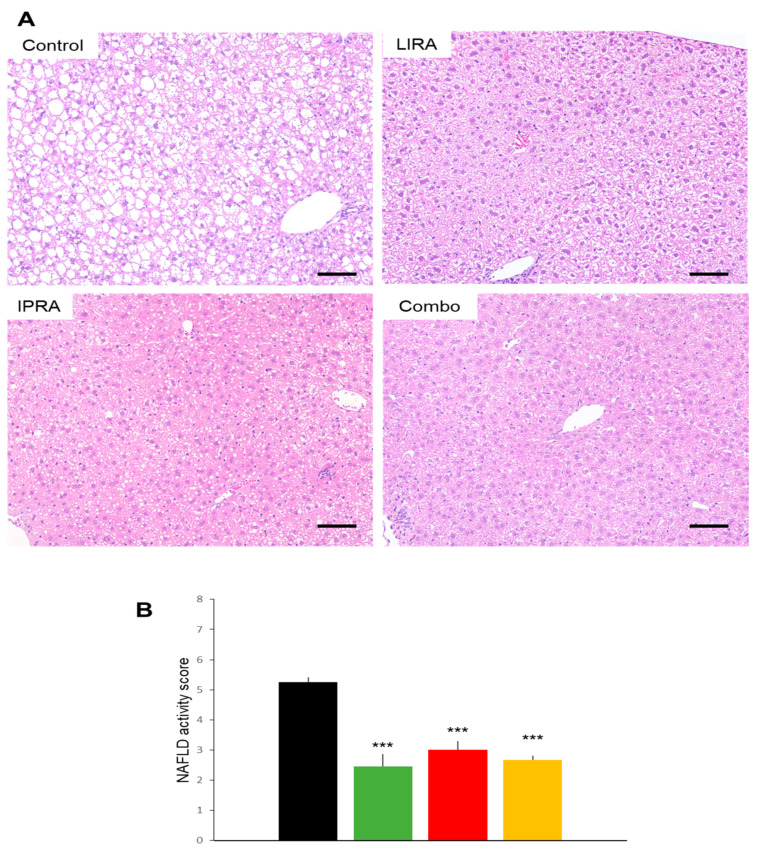
Effects of treatments with liraglutide and/or ipragliflozin on livers in DIO mice. Representative liver sections (**A**) and NAFLD activity score (**B**) of vehicle-treated DIO mice and DIO mice treated with liraglutide and/or ipragliflozin. Data represent mean ± SE, *n* = 6, *** *p* < 0.001 vs. Control. Scale bars = 200 μm.

**Figure 4:**
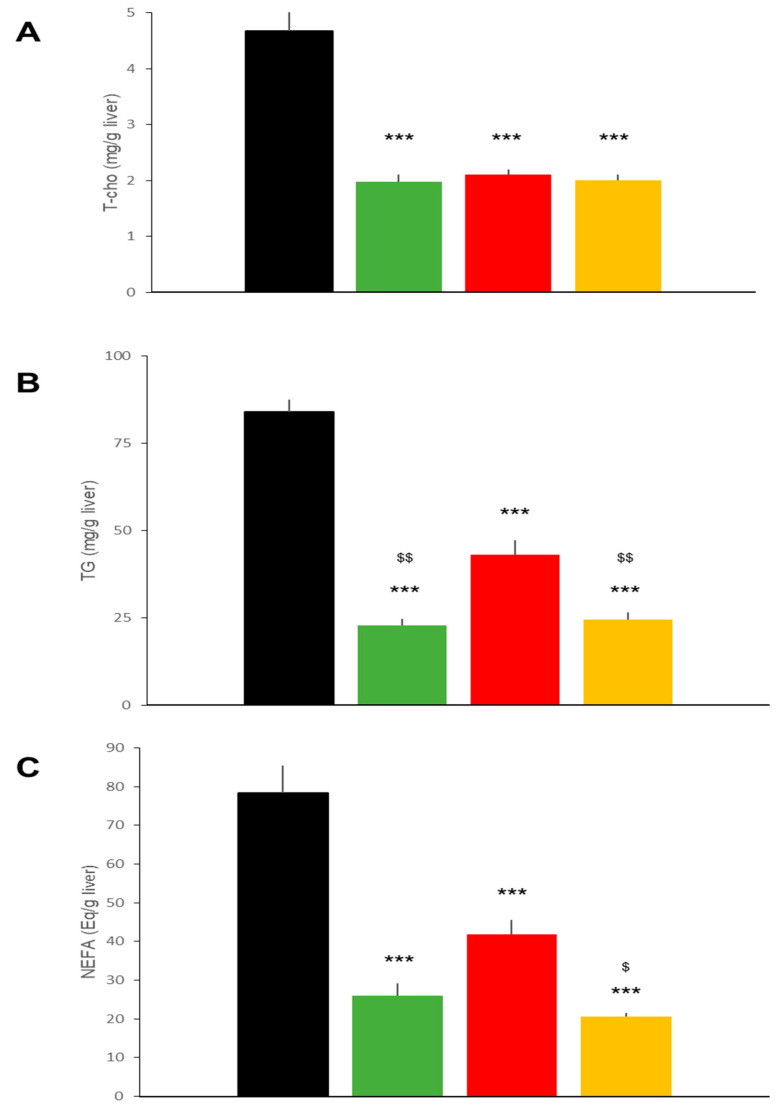
Effects of treatments with liraglutide and/or ipragliflozin on hepatic lipid contents in DIO mice. Hepatic total cholesterol (**A**), triglyceride (**B**), and non-esterified fatty acid (**C**) contents of vehicle-treated DIO mice and in mice treated with liraglutide and/or ipragliflozin. Data represent mean ± SE, *n* = 6, *** *p* < 0.001 vs. Control; $ *p* < 0.05, $$ *p* < 0.01 vs. IPRA group.

Hepatic histology (Figure 3A) in the DIO mice revealed that liraglutide and ipragliflozin, individually and in combination, ameliorated fatty liver as demonstrated by a diminished non-alcoholic fatty liver disease (NAFLD) activity score [11] to similar degrees (Figure 3B). Hepatic lipid accumulation was also reduced in the three treatment groups, though ipragliflozin alone showed slightly weaker effects than the other two regimens (Figure 3B). Thus, hepatic TG in mice of the LIRA and the Combo groups were significantly lower than those in IPRA group mice (Figure 4).

**Figure 5:**
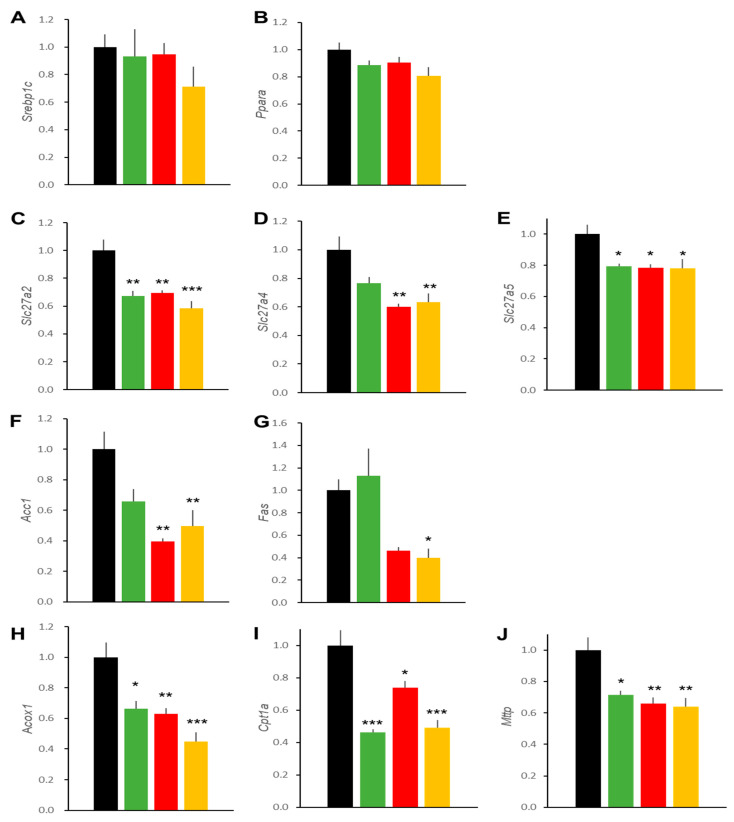
Effects of treatments on hepatic expressions of genes related to lipid metabolism in DIO mice. Expressions of two master regulator genes of hepatic lipid metabolism: *Srebp1c* (**A**) and *Ppara* (**B**). Genes important for fatty acid uptake: *Fatp2* (**C**), *Fatp4* (**D**), and *Fatp5* (**E**). Genes important for fatty acid synthesis: *Acc1* (**F**) and *Fas* (**G**), and for fatty acid oxidation: *Acox1* (**H**) and *Cpt1a* (**I**). Gene important for TG excretion: *Mttp* (**J**). Data represent mean ± SE, *n* = 6, * *p* < 0.05, ** *p* < 0.01, *** *p* < 0.001 vs. Control group.

In order to gain insight into the molecular basis for the altered lipid accumulation in livers of DIO mice treated with liraglutide and/or ipragliflozin, we determined the transcript levels of several genes important for hepatic lipid metabolism (Figure 5). First, master regulator transcription factors of hepatic lipid metabolism, *Srebp1c* and *Ppara*, showed no differences among the four groups. Fatty acid transporter isoforms, *Slc27a2*, *4*, and *5*, the only exception being *Slc27a4* in the LIRA group, were reduced by these treatments. Genes important for fatty acid generation (*Acc1* and *Fas*) and fatty acid degradation (*Acox1* and *Cpt1a*) were reduced as well. A gene encoding microsomal triglyceride transfer protein (*Mttp*), which is important for TG excretion, was also reduced.

### 2.4. Effects on BW and Glycemia in db/db Mice

**Figure 6:**
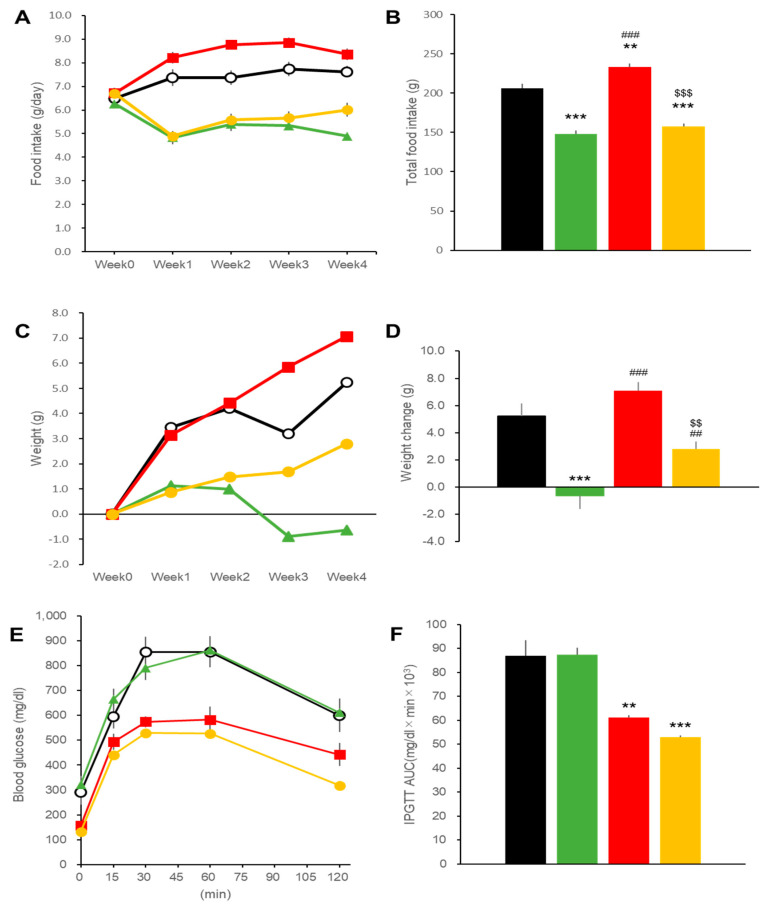
Effects of treatments with liraglutide and/or ipragliflozin on food intakes, body weight, and glycemic control in db/db mice. Time courses (**A**) and total amounts (**B**) of food intake in vehicle-treated db/db mice (black line and bar) and in db/db mice treated with liraglutide (green), ipragliflozin (red,) and the combination regimen (orange). Time courses (**C**) and final changes (**D**) in body weight in db/db mice treated with liraglutide and/or ipragliflozin. Blood glucose excursions during intraperitoneal glucose tolerance tests (**E**) and AUC (**F**) calculated from (**E**). Data represent mean ± SE, *n* = 6, ** *p* < 0.01, *** *p* < 0.001 vs. Control group; $$ *p* < 0.01, $$$ *p* < 0.001 vs. IPRA group; ## *p* < 0.01, ### *p* < 0.001 vs. LIRA group. Week0 was 8 weeks of age.

Daily food intakes were around 6.5 g/day in the db/db mice, and approximately 2.5-fold greater than those for the DIO mice. Food intakes were decreased in the LIRA and Combo groups (Figure 6A,B), while the IPRA group showed increased food intakes as compared to the Control db/db mice. Body weights at 8 weeks of age were 38.4 ± 0.4 g (mean ± SEM, *n* = 24), comparable to those of the DIO mice at 12 weeks. Body weight gain was not observed, and the LIRA group mice weighed significantly less than those of the other groups. Mice in the IPRA group showed greater weight gain than the Control group of mice. Combination therapy had an intermediate effect on body weight (Figure 6C,D).

Effects on glycemic excursions during OGTT in the db/db mice were different from those in the DIO mice. In db/db mice, liraglutide exerted no beneficial effects on glycemia. In contrast, ipragliflozin alone produced substantial decreases in glycemic excursion whether administered alone or in combination with liraglutide (Figure 6E,F).

### 2.5. Pancreatic Effects in db/db Mice

**Figure 7:**
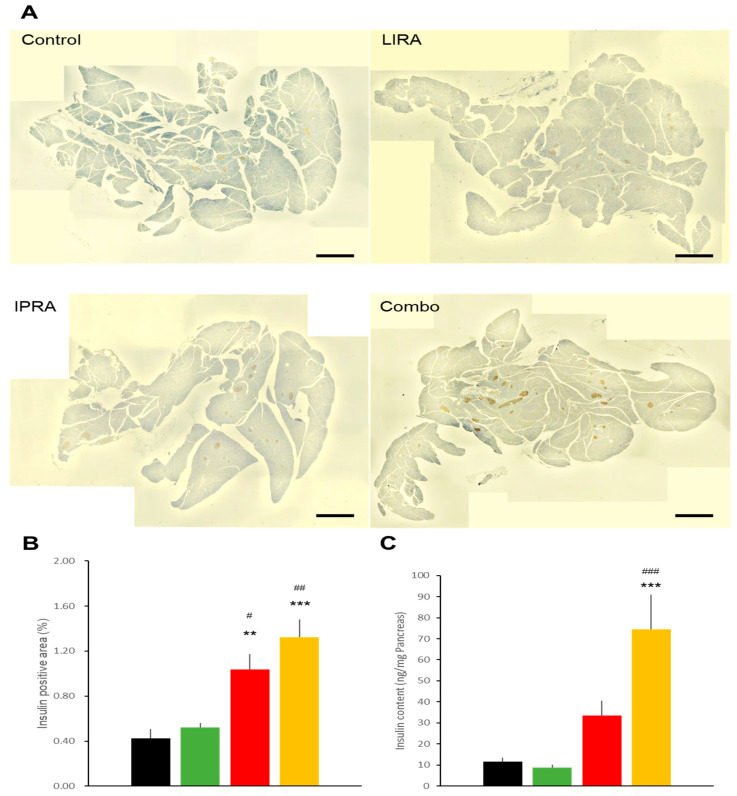
Effects of treatments with liraglutide and/or ipragliflozin on pancreatic islets in db/db mice. Representative pancreatic sections (**A**), insulin positive areas (**B**), and insulin contents (**C**) of vehicle-treated db/db mice and in db/db mice treated with liraglutide and/or ipragliflozin. Data represent mean ± SE, *n* = 6, ** *p* < 0.01, *** *p* < 0.001 vs. control group; # *p* < 0.05, ## *p* < 0.01, ### *p* < 0.001 vs. LIRA group. Scale bars = 500 μm. An additional cohort of db/db mice was established, since insulin positive areas and insulin content cannot be obtained simultaneously from one mouse. Effects on glycemic control in this additional cohort did not differ from other cohorts.

Fasting plasma insulin levels were increased in the IPRA and Combo groups (Table 2). Glucagon levels were not statistically different among the treatment groups. As demonstrated in Figure 7A,B, liraglutide alone exerted no beneficial effects on the insulin-positive area in the db/db pancreas. In db/db mice of the IPRA and Combo groups, the insulin-positive area was greater than in the Control and LIRA group mice. Similarly, pancreatic insulin content tended to be increased in mice of the IPRA group and was significantly increased in Combo group mice (Figure 7C). Pancreatic insulin content in the group receiving combination therapy was twice that in the mice treated with ipragliflozin alone, although the difference was slightly short of statistical significance (*p* = 0.0568).

### 2.6. Hepatic Effects in db/db Mice

Although plasma AST levels were similar, ALT levels were lower in the LIRA and Combo group mice than in the Control group (Table 2). Plasma TG levels were also significantly reduced in the LIRA and Combo groups (Table 2). 

**Figure 8:**
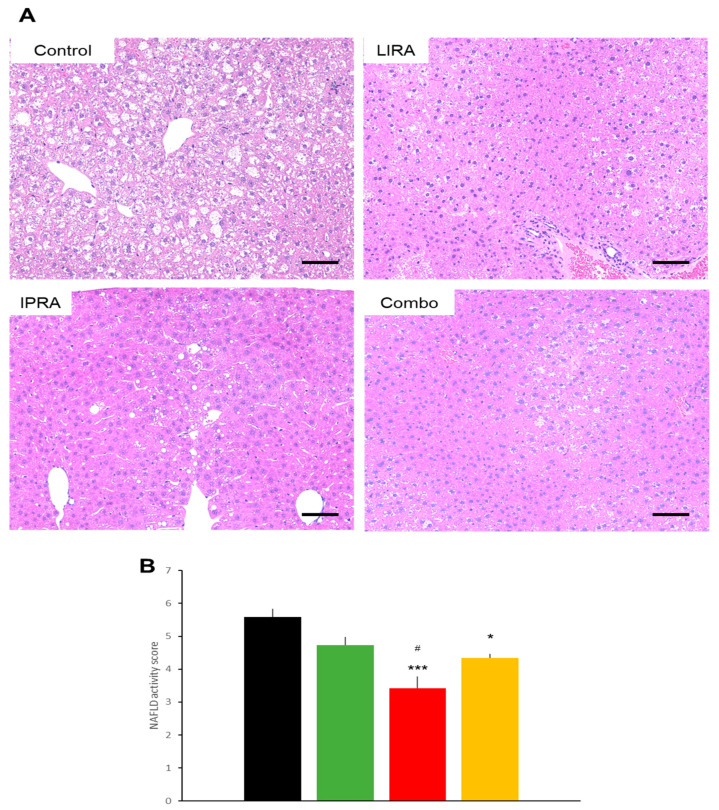
Effects of treatments with liraglutide and/or ipragliflozin on livers of db/db mice. Representative liver sections (**A**) and NAFLD activity score (**B**) of vehicle-treated db/db mice and of db/db mice treated with liraglutide and/or ipragliflozin. Data represent mean ± *n* = 6, * *p* < 0.05, and *** *p* < 0.001 vs. control group, # *p* < 0.05 vs. LIRA group. Scale bars = 100 μm.

**Figure 9:**
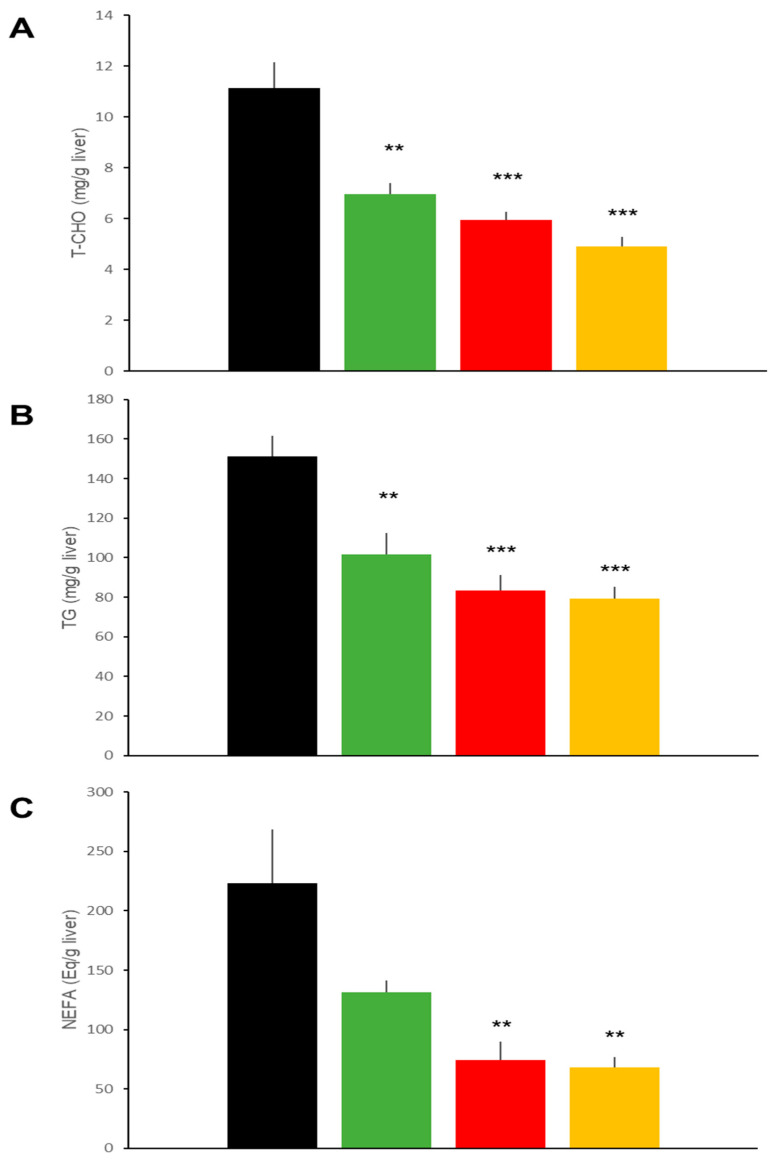
Effects of treatments with liraglutide and/or ipragliflozin on hepatic lipid contents in db/db mice. Hepatic total cholesterol (**A**), TG (**B**), and non-esterified fatty acid (**C**) contents of vehicle-treated db/db mice and of db/db mice treated with liraglutide and/or ipragliflozin. Data represent mean ± SE, *n* = 6, ** *p* < 0.01, and *** *p* < 0.001 vs. control group.

Hepatic histology in the db/db mice showed that liraglutide tended to reduce the NAFLD activity score [11], although the difference was not statistically significant. The analysis showed that ipragliflozin, individually and in combination with liraglutide, ameliorated the score (Figure 8). Hepatic lipid accumulation was also reduced by the three treatments (Figure 9). Liraglutide appeared to have less of an effect on hepatic lipid accumulation but there were no statistically significant differences among the treatment regimens.

**Figure 10:**
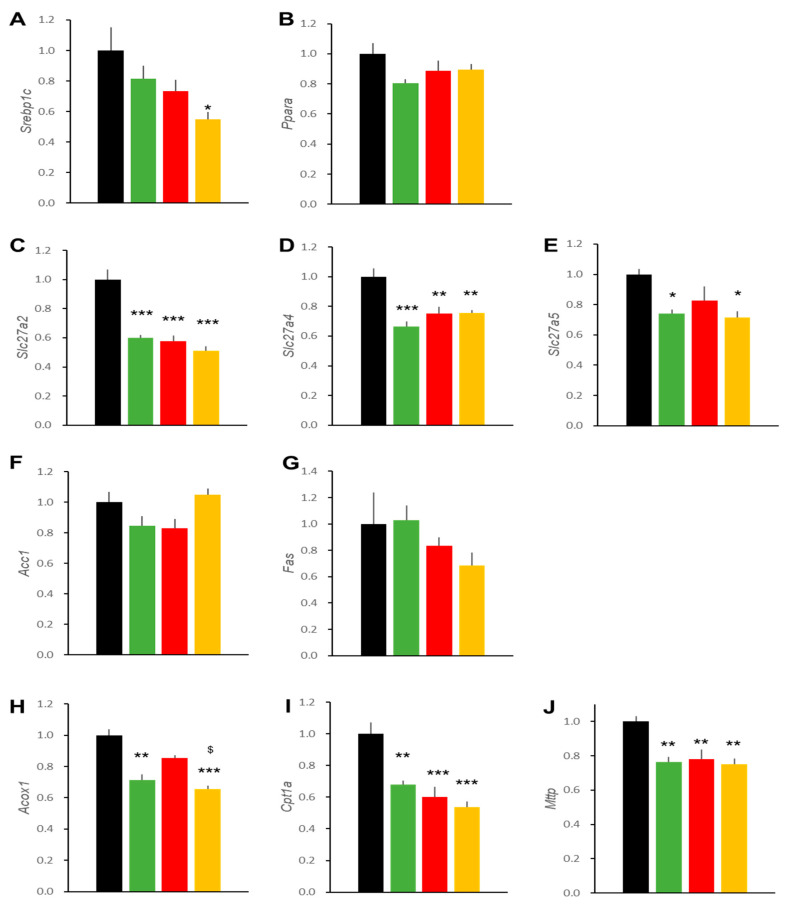
Effects of treatments on hepatic expressions of genes related to lipid metabolism in db/db mice. Expression of two master regulator genes of hepatic lipid metabolism: *Srebp1c* (**A**) and *Ppara* (**B**). Genes important for fatty acid uptake: *Fatp2* (**C**), *Fatp4* (**D**), and *Fatp5* (**E**). Genes important for fatty acid synthesis: *Acc1* (**F**) and *Fas* (**G**), and for fatty acid oxidation: *Acox1* (**H**) and *Cpt1a* (**I**). Gene important for TG excretion: *Mttp* (**J**). Data represent mean ± SE, *n* = 6, * *p* < 0.05, ** *p* < 0.01, and *** *p* < 0.001 vs. control group. $ *p* < 0.05 vs. IPRA group.

Transcript levels of several genes important for hepatic lipid metabolism were also analyzed in the livers of db/db mice treated with liraglutide and/or ipragliflozin, (Figure 10). A key regulator of hepatic lipid metabolism, *Srebp1c*, tended to be reduced by treatment with liraglutide or ipragliflozin and was significantly reduced by combination therapy with these two agents. Fatty acid transporter isoforms, *Slc27a2, 4*, and *5*, were also reduced. Effects of the two agents were neither additive nor synergistic. While genes important for fatty acid generation (*Acc1* and *Fas*) were unchanged, those involved in fatty acid degradation (*Acox1* and *Cpt1a*) were reduced. Finally, a gene encoding microsomal triglyceride transfer protein (*Mttp*) was also reduced.

## 3. Discussion

Directly comparing different models of T2DM clarified the characteristics of SGLT2i (ipragliflozin) and GLP-1RA (liraglutide) agents. Effects of these drugs, either singly or in combination, on glycemic control differed between these models representing the early and advanced stages of T2DM. GLP-1RA was more effective than SGLT2i in the DIO mice, while effects on glycemia in the db/db mice showed the opposite tendency. In fact, liraglutide had no effect on glycemic control in the db/db mice. 

Effects on body weight and glycemic control showed good correlations among DIO mice receiving four different treatment regimens, suggesting that energy balance might be among the major factors contributing to glycemic control in DIO mice. In contrast, body weight changes did not correlate to glycemic control in the db/db mice. Instead, glycemic control showed an inverse correlation with effects on β-cells mass in all groups of db/db mice.

Pancreatic insulin content in the DIO mice (45 ng/mg pancreas) was much higher than that in the db/db mice (10 ng/mg), confirming that DIO mice are a model of early-stage diabetes, while db/db mice serve as an advanced-stage model. With minimal impairment of β-cells, liraglutide exerted its effect on glycemic control in DIO mice. However, the GLP-1RA did not ameliorate the severe impairment in β-cells and thus had no beneficial effect on glycemic control, supporting the notion that starting GLP-1RA treatment as early as possible would maximize the clinical benefits [12,13]. At the beginning of the treatments, the blood insulin level was around 6 ng/mL in the db/db mice. As shown in Table 2, after a 4-week treatment, blood insulin levels dropped by at least half in the Control and LIRA groups of mice, indicating that the GLP-1RA does not prevent the accelerated β-cell loss characteristic of db/db mice. In contrast, beneficial effects of ipragliflozin on db/db β-cells were prominent. Surprisingly, the pancreatic insulin content in the mice treated with liraglutide together with ipragliflozin was almost double that of the mice administered ipragliflozin alone, although the difference failed to reach statistical significance (*p* = 0.0568). These data suggest that in the absence of glucose toxicity, GLP-1RAs exert stimulatory effects on insulin production and possibly on β-cell proliferation. 

Miller and colleagues reported the effects of combined treatment with dapagliflozin and liraglutide on β-cells in DIO mice who were also administered streptozotocin (β-cell toxin) [14]. The severity of the glycemic abnormality in their mice might be intermediate between those in our two models. They found that liraglutide increased pancreatic insulin content but that dapagliflozin reduced it. There were no combined effects in mice treated with dapagliflozin and liraglutide as compared to the effects on mice administered these drugs individually. The reason for the discrepancies in results between their study and ours, especially the different effects of SGLT2i, are currently unknown. The stage of diabetes may well have an impact on treatment outcomes. 

In this study, we observed no changes in plasma glucagon levels with liraglutide and/or ipragliflozin treatments in either the DIO or the db/db mice, although glucagon levels in the db/db mice in the Combo group tended to increase. The effect of SGLT2is on glucagon secretion is a matter of debate [15]. Earlier studies [16,17], including ours [18] demonstrated that SGLT2is increase plasma glucagon levels. However, glucagon levels in these studies were evaluated with less reliable conventional methods. New sandwich enzyme-linked immunosorbent assays with improved specificities for glucagon became available [19], and we have used such a method in this study. Furthermore, detailed analysis indicated that SGLT2 is not expressed in α-cells [20], suggesting that the mechanisms of SGLT2is on glucagon secretion are unknown and the effects, if any, of SGLT2is on plasma glucagon levels are indirect. Further studies are needed to elucidate the roles of glucagon in the therapeutic effects of SGLT2is. 

Both liraglutide and ipragliflozin ameliorated NAFLD in the DIO as well as the db/db mice. The etiology of NAFLD differed between these two models, however. In DIO mice, HFD induced NAFLD, while in db/db mice, despite being fed normal chow, hyperphagia induced NAFLD. Serum lipid profiles and their changes in response to treatments also differed between the two models. As previously reported in DIO mice treated with SGLT2i [21,22] or GLP-1RA [23,24], total cholesterol rather than TG was reduced by ipragliflozin and liraglutide, both alone and in combination. On the other hand, cholesterol was markedly reduced by these agents in db/db mice, as previously reported [25,26]. Nonetheless, hepatic accumulations of cholesterol, TG, and fatty acids were similarly reduced by liraglutide and/or ipragliflozin in both models. It should be noted that liraglutide reduced lipid accumulation, despite having no effect on glycemic control in our db/db mice. Several mechanisms by which SGLT2i and GLP-1RA exert beneficial effects on fatty liver disease have been proposed [27]. Weight loss is the first possibility. This mechanism may be applicable to our DIO but not our db/db mice. In the present study, the NAFLD activity score and lipid accumulation were clearly observed in the db/db mice treated with ipragliflozin despite increased food intake and weight gain. In these mice, other mechanisms must be operating, such as forced excretion of glucose into the urine and the subsequent utilization of hepatic lipid as an energy source [28]. 

Effects on the expressions of genes related to liver lipid synthesizing and oxidizing enzymes have also been postulated. SGLT2 is not expressed in hepatocytes and its effects were attributed to reduced blood glucose levels and the subsequent amelioration of insulin resistance, which might have altered the expressions of genes regulating hepatic lipid metabolism. The existence of GLP-1 receptors in hepatocytes is a matter of debate [29]. Thus, the effects of liraglutide on lipid accumulation in hepatocytes and gene expressions may not represent direct actions of liraglutide; instead, these effects might be exerted either through other cells residing in the liver or via humoral factors or the neural network. Several studies have analyzed expressions of lipid metabolism genes in response to GLP-1RAs and SGLT2is in the liver. The results are not consistent, possibly due to differences in food intake, strains of mice, and protocols including treatment periods. Typically, fatty liver can be ameliorated by reducing lipid uptake and the suppression of de novo lipid synthesis as well as increased lipid oxidation and enhanced lipid excretion, actions orchestrated by Srebp1c and PPARα [30]. In our present study, we detected no alteration, a tendency to be decreased, or significant decreases in genes related to lipid uptake, lipid synthesis, oxidation, and excretion. Liraglutide-induced decreases in *Acox1* [31] and *Cpt1a* [32] transcripts have been reported by other investigators, but reductions in lipid oxidizing genes and lipid excretion genes do not appear to be mechanistically consistent with our present understanding. These changes could result from improvement in fatty liver pathology through reduced nutrient load and increased lipid consumption after 4-week treatment periods. Time courses of the expressions of these genes should merit further detailed studies.

This study has several limitations. First, we administered liraglutide at a dose of 300 μg/kg/day, which was within the dose ranges used in previous studies [12,13,24,25,26] but higher than clinical doses. Second, paired feeding was not conducted. This caused an imbalance of not only calorie intake but also amounts of ipragliflozin, since it was given in the food. We observed a 30% reduction in food intake in the LIRA and Combo groups as compared to the IPRA group, resulting in the ipragliflozin doses being 30% lower in the Combo group than in the IPRA group. Caution must be exercised when interpreting the results. 

As discussed above, although ipragliflozin doses were somewhat lower in mice treated with a combination regimen including liraglutide than in those given SGLT2i alone, beneficial effects on β-cells and the liver as well as on glycemic control were observed in the Combo group as in the IPRA group. In addition, parameters such as *Fas* transcript in the DIO mice and *Srebp1c* transcript in the db/db mice were reduced only in the mice given the combination therapy. Therefore, the SGLT2i plus GLP-1RA combination is a promising strategy for diabetes, especially for the advanced stage of diabetes with NAFLD.

## 4. Materials and Methods

### 4.1. Animals

Mice were kept one per cage at a room temperature of 22 ± 2 °C, with lights on/off at 8AM/8PM. Male DIO model mice were purchased from Charles River Japan at 14 weeks of age. They were C57BL/6J mice and had been fed, starting at 4 weeks of age, a 60% high fat diet (D12492, Research Diets Inc, New Brunswick, NJ, USA). They had been fed a similar 60% HFD from Oriental Kobo Japan (HFD-60: 62.2% fat, 18.2% protein, 19.6% carbohydrate) in our animal facility. After 2 weeks, the mice were randomly divided into four groups: mice given no treatment (Control group), mice treated with liraglutide (LIRA group), mice treated with ipragliflozin (IPRA group), and mice treated with the combination of liraglutide and ipragliflozin (Combo group). The diet and treatments lasted for 4 weeks.

Mice in the LIRA group were treated with a daily intraperitoneal injection of liraglutide (300 μg/kg). This dose was established by previous studies [11,12,17,18,19]. Mice in the IPRA group were fed a diet containing ipragliflozin (0.002%). Since the DIO mice consumed on average 2.5 g food/day at 16 weeks, this corresponded to ipragliflozin administration of 1.5 mg/kg/day. For the db/db mice, dietary intake corresponded to 6.5 mg/kg/day, and ipragliflozin concentration was 0.001%. The mice in this group received intraperitoneal saline administration daily. Mice in the Combo group had been treated with both liraglutide and ipragliflozin. Mice in the Control group had been also given daily intraperitoneal saline administrations. Male leptin receptor deficient C57BL/6^+Lepr<db>/+Lepr<db>^ mice (db/db) were purchased at 6 weeks of age from Charles River Japan. They were fed standard chow. After 2 weeks of acclimation, drug treatments were started. 

For both models, intraperitoneal glucose tolerance tests were conducted at the beginning and after the 4-week treatment period. The mice were then anesthetized and killed by blood sampling from the abdominal aortas. The liver, kidneys, pancreas, and brain were removed and immediately frozen with dry ice or immersed in 4% paraformaldehyde. The pancreases were dissolved in acid ethanol (0.7 N HCl/ethanol 25:75) and insulin was thereby extracted. 

### 4.2. Histochemical Studies

Liver specimens were fixed with 4% paraformaldehyde and embedded in paraffin. The liver sections were then stained with hematoxylin and eosin, for viewing with a BZ-X710 microscope (Keyence, Osaka, Japan). Steatohepatitis was evaluated based on the NAFLD Activity Score [10]. 

Paraffin-embedded pancreatic sections were stained with anti-insulin antibody (Abcam, Cambridge, UK). The sections were viewed with a microscope (BX51, Olympus, Tokyo, Japan), the images were tiled using an e-Tiling software program (Mitani Co., Tokyo, Japan), and insulin-positive areas were analyzed using WinROOF software (Mitani). 

### 4.3. Measurements of Serum Parameters, Hepatic Lipids, and Pancreatic Insulin Content

Serum AST, ALT, ALP, and TG, as well as total cholesterol, were measured using agents from Fujichemical Wako (Wako, Osaka, Japan). For the measurement of hepatic lipid content, frozen liver specimens were homogenized using a homogenizer Physcotron NS-310E3 (Microtec, Chiba, Japan) and lipids were extracted using the Folch method [33], followed by measurement using the same agents as above. Pancreatic insulin content was measured using an ELISA (Mercodia, Uppsala, Sweden) after extraction in acid ethanol for 48 h. 

### 4.4. Quantitative Real Time Reverse Transcription PCR

Frozen liver tissues were homogenized using a homogenizer (Physcotron NS-310E3) and total RNA was extracted with an RNAeasy kit (Qiagen, Redwood City, CA, USA). After synthesizing cDNA pools using a RivaTra Ace kit (Toyobo, Tokyo, Japan), mRNA expressions were analyzed with the primers listed in Table S1 using LightCycler96 (Roche, Basal, Switzerland). 

### 4.5. Statistical Analysis

Results are expressed as means ± SE. Statistical significance was assessed using ANOVA. All statistical analyses were performed with EZR [34].

## Figures and Tables

**Table 1:** Plasma biochemical parameters in DIO mice after drug treatment.

Parameter	Control	LIRA	IPRA	Combo
Insulin (ng/mL)	0.40 ± 0.04	0.54 ± 0.14	0.46 ± 0.10	0.37 ± 0.11
			
Glucagon (pmol/L)	11.3 ± 0.8	11.0 ± 0.3	9.3 ± 1.1	11.0 ± 0.8
			
AST (IU/L)	57.0 ± 5.3	46.8 ± 3.2	60.0 ± 8.9	56.0 ± 4.4
			
ALT (IU/L)	36.7 ± 6.6	13.2 ± 0.50	22.2 ± 1.7	18.3 ± 2.5
		**		*
T-cho (mg/dL)	164.7 ± 3.9	130.3 ± 2.7	152.5 ± 0.9	120.0 ± 2.4
		*** $$$	*	*** $$$
TG (mg/dL)	71.0 ± 5.5	68.3 ± 8.5	62.5 ± 6.6	58.7 ± 2.9
			

Data represent means ± standard error, *n* = 6. * *p* < 0.05, ** *p* < 0.01, *** *p* < 0.001 vs. control; $$$ *p* < 0.001 vs. ipragliflozin, one-way ANOVA.

**Table 2:** Plasma biochemical parameters in db/db mice after drug treatment.

Parameter	Control	LIRA	IPRA	Combo
Insulin (ng/mL)	2.98 ± 0.16	1.90 ± 0.32	4.64 ± 0.60	5.34 ± 0.71
			#	*,##
Glucagon (pmol/L)	12.5 ± 2.4	13.9 ± 3.2	17.2 ± 3.1	24.9 ± 2.4
				*p* = 0.0600 vs. control
AST (IU/L)	66.2 ± 3.9	58.7 ± 1.84	70.5 ± 4.48	51.8 ± 2.53
				$$
ALT (IU/L)	73.5 ± 5.82	44.0 ± 2.2	65.7 ± 6.1	40.0 ± 2.9
		**,$$		***,$$
T-cho (mg/dL)	127.5 ± 6.76	109.8 ± 5.13	131.3 ± 3.6	113.0 ± 1.5
		$		
TG (mg/dL)	244.2 ± 20.0	147.2 ± 17.3	187.2 ± 28.8	107.8 ± 12.1
		*		**

Data represent means ± standard error, n = 6. * *p* < 0.05 vs. control; ** *p* < 0.01 vs. control; *** *p* < 0.001 vs. control; # *p* < 0.05, ## *p* < 0.01 vs. liraglutide; $ *p* < 0.05, $$ *p* < 0.01 vs. ipragliflozin, one-way ANOVA.

## Data Availability

The data presented in this study are available on request from the corresponding authors.

## Supplementary Materials

The following is available online at https://www.mdpi.com/article/10.3390/ijms222111463/s1.
