# Supplementary material for: Effects of GLP-1RA and SGLT2i, Alone or in Combination, on Mouse Models of Type 2 Diabetes Representing Different Disease Stages
Source: Int J Mol Sci. 2021 Oct 25;22(21):11463. doi: 10.3390/ijms222111463 (PMC8583813; doi:10.3390/ijms222111463)
Supplement: Supplementary file 1 [file ijms-22-11463-s001.zip › ijms-1425564-supplementary.pdf]

Table S1

Primers used for qRT-PCR analysis of genes important for hepatic lipid metabolism

Srebp1c	Forward primer	TACAGCGTGGCTGGGAAC
	Reverse primer	CTCCCTGTCTCCGTCAGC
Ppara	Forward primer	CTGAGACCCTCGGGGAAC
	Reverse primer	AAACGTCAGTTCACAGGGAAG
Slc27a2	Forward primer	GCGTGCCTCAACTACAACATT
	Reverse primer	CCTCCTCCACAGCTTCTTGT
Slc27a4	Forward primer	CTTGCCTGAGCTGCACAA
	Reverse primer	GCGGGTCTTTCACAACAGAT
Slc27a5	Forward primer	CTGCGGTACTTGTGTAACGTCC
	Reverse primer	TCCGAATGGGACCAAAGCGTTG
Acc1	Forward primer	GCGTCGGGTAGATCCAGTT
	Reverse primer	CTCAGTGGGGCTTAGCTCTG
Fas	Forward primer	TACAGCGTGGCTGGGAAC
	Reverse primer	CTCCCTGTCTCCGTCAGC
Acox1	Forward primer	ATCAGGGCACCACTGCTC
	Reverse primer	CCAAGCCTCGAAGATGAGTT
Cpt1a	Forward primer	GACTCCGCTCGCTCATTC
	Reverse primer	TCTGCCATCTTGAGTGGTGA
Mttp	Forward primer	GCCCAACGTACTTCTAATTTATGG
	Reverse primer	TGCTGGCCAACACGTCTA

Primers used for qRT-PCR analysis of genes important for hepatic lipid metabolism
